# Kawasaki Disease Presenting Uncommonly with Torticollis: A Case Report

**DOI:** 10.1002/ccr3.70796

**Published:** 2025-08-22

**Authors:** Hassan Mottaghi Moghaddam Shahri, Faeze Keihanian, Mohammad Hassan Nezafati, Mohammad Saeid Sasan

**Affiliations:** ^1^ Pediatric Department Faculty of Medicine, Mashhad University of Medical Sciences Mashhad Iran; ^2^ Cardiology Department Faculty of Medicine, Mashhad University of Medical Sciences Mashhad Iran; ^3^ Cardiac Surgery Department Faculty of Medicine, Mashhad University of Medical Sciences Mashhad Iran

**Keywords:** coronary artery aneurysm, coronary artery bypass graft surgery, Kawasaki disease, torticollis

## Abstract

Kawasaki disease (KD) is an acute, febrile, systemic inflammatory disorder affecting children. A subset of KD patients, often infants or older children, does not meet the classic diagnostic criteria. In such cases, delayed recognition of atypical or incomplete KD can increase the risk of coronary artery complications. We report the case of a previously healthy 7‐year‐old girl who initially presented with isolated cervical adenitis, without fever, conjunctivitis, or extremity edema. She was initially treated for an infectious disease. After 10 days, additional clinical features emerged, raising suspicion for atypical KD. Subsequent evaluation revealed giant coronary artery aneurysms, necessitating coronary artery bypass graft surgery (CABG), aneurysmectomy, and aneurysmorrhaphy to prevent complications such as aneurysm rupture. She remained asymptomatic during a two‐year follow‐up period. This case highlights the importance of maintaining a high index of suspicion for KD in children presenting with lymphadenopathy and unusual clinical features, especially when empirical antibiotic therapy is ineffective. Early recognition and treatment are crucial to prevent serious cardiac complications.

**Summary**

- In cases of isolated torticollis in children, it is important to emphasize considering Kawasaki disease (KD)—specifically atypical or incomplete KD—as part of the differential diagnosis, alongside more common causes.

**Abbreviations**
CABG: coronary artery bypass graft surgery
CT: computed tomography
EF: ejection fraction
IVIG: Intravenous immune globulin
KD: Kawasaki disease
LAD: left anterior descending artery
LIMA: Left internal mammary artery
LV: left ventricular
MR: mitral regurgitation
RCA: right coronary artery

## 1 Introduction

Kawasaki disease (KD) is a systemic vasculitis primarily affecting medium‐sized arteries, especially the coronary arteries, and is one of the most common vasculitis in childhood [1]. It can lead to severe adverse events, morbidity, and mortality if not suitably managed. The majority of patients are less than 5 years old, and the male to female ratio is about 1.3–1.6:1 [2, 3]. It is a prevalent disorder all over the world with higher predisposition in Asian ethnic groups. Typically, KD can be diagnosed by fever persisting for at least 5 days and the presence of at least four of the following principal features: [1] changes in the extremities; [2] polymorphous exanthema; [3] bilateral bulbar conjunctival injection without exudates; [4] changes in the lips and oral cavity; and [5] cervical lymphadenopathy (> 1.5 cm diameter). Exclusion of other diseases with similar symptoms is also necessary [4]. When involving the cardiovascular system, patients may present with myocarditis, pericarditis, coronary artery aneurysm, and aortic root dilatation [2, 5]. Incomplete KD is characterized by a longer fever duration, younger age of onset, and higher incidence of coronary artery disease compared with complete KD [6]. Owing to systemic involvement, KD shows significant variability in multiple organ systems, and early diagnosis in patients with atypical KD or in those with uncommon manifestations is difficult. With the widespread use of computed tomography (CT), there is an increasing number of reports of KD with retropharyngeal edema and enlarged cervical adenopathy [7, 8]. Progressing into shock is uncommon; although some studies have reported patients with KD and shock syndrome [9, 10]. In this report, in addition to interesting echocardiographic and angiographic findings, diagnostic criteria, cardiovascular involvement, and risk factors have also been reviewed in KD patients.

## 2 Case History/Examination

A 7‐year‐old girl presented with isolated cervical adenitis with no fever, conjunctivitis, or edema. She had no history of previous disease and had appropriate development. She was treated for suspected infectious disease with cephalexin. Because of no suitable response, cefotaxime and clindamycin were started. Pain and swelling over the right side of her neck with torticollis remained. There was no history of trauma to the head and neck. The child was immunized for her age as per the national vaccination program. After one week, fever started, and due to high grade fever (39–40 C), evidence of systemic inflammation, fatigue, malaise, ankle, hand, and elbow pain, and arthritis, she was admitted to our hospital.

**FIGURE 1:**
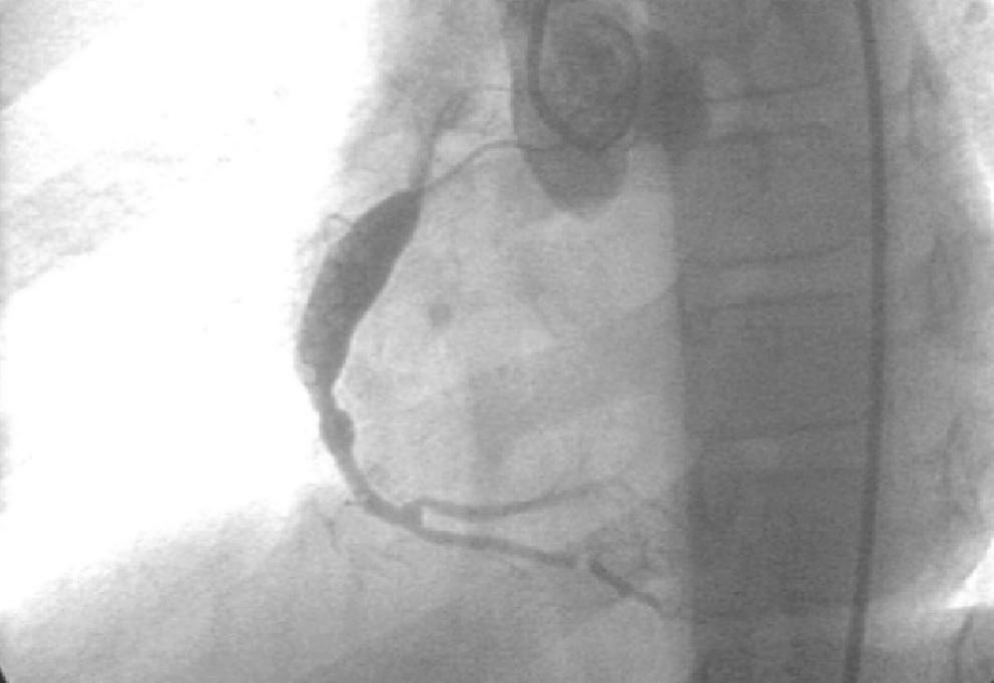
Coronary angiography before CABG showing giant aneurysm in right coronary artery.

She was ill, and her head was tilted to the right with chin rotation to the left. Her vital signs at the time of admission were: heart rate: 85/min, respiratory rate: 25/min, blood pressure: 110/80 mmHg, and temperature: 37.2°C. After 10 days, other classic signs of KD were revealed. Physical examination found erythematous and congested throat, palpable lymph nodes, normal breath sounds, no audible heart murmur, and no skin rash. Initial laboratory data demonstrated pyuria, a white blood cell count of 23.4 × 10^9^/L, and an elevated CRP level of 103 mg/L and platelet count 620,000/mm^3^. The hemoglobin was 12.3 mg/dL, albumin was 3.3 g/dL, AST and ALT were 35 and 45 U/L, respectively. She received intravenous antibiotic therapy (cefotaxime and vancomycin). Due to the lack of IVIG in the pediatric infectious services, she was treated with steroids, and aspirin was started at an anti‐inflammatory dose of 80 mg/Kg in four divided doses. Her electrocardiogram had no significant pathologic changes and cardiac troponin I was negative. Two‐dimensional echocardiography on day 14 showed giant coronary artery aneurysms, left ventricular (LV) dyskinesia, and mitral regurgitation (MR). Catheterization and selective coronary angiography on day 16 of the disease showed giant aneurysmal dilatation of both coronary arteries, clot formation in coronary arteries, and LV dysfunction (Figure 1).

## 3 Differential Diagnosis

Torticollis is a clinical symptom determined by a lateral head tilt and chin rotation into the opposite side of the tilt. There are several etiologies causing torticollis. Because our case is not in infancy, some of the differential diagnoses were excluded, like congenital muscular torticollis. In older childhood, like our case, the most common cause is rotation and displacement of the atlantoaxial axis due to trauma or inflammation. Other diagnoses are included: retropharyngeal abscesses and pyogenic cervical spondylitis. Posterior fossa tumors may intermittently mimic torticollis besides neurologic symptoms. Other rare etiologies are: benign and malignant neoplasms of the upper cervical spine and cervical dystonia [11].

## 4 Conclusion and Results (Outcome and Follow‐Up)

**FIGURE 2:**
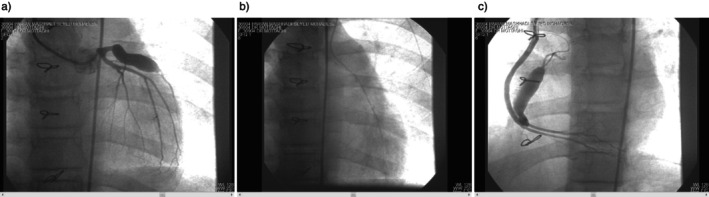
Postoperative angiography. (a) LAD aneurysm, (b) LIMA to LAD, (c) venous graft to RCA.

In our patient, coronary artery bypass graft surgery (CABG; LIMA to LAD and venous graft to RCA), aneurysmectomy, and aneurysmorrhaphy were done by the cardiac surgeon at the second month of disease because of prevention from adverse events like rupture. Although LV function improved and she had no complaints, warfarin therapy continued due to aneurysmal LAD. A follow up angiography was also done (Figure 2). She is followed up until now (2 years, every 6 months) and has no sequelae.

## 5 Discussion

Attention to the initial atypical manifestation of KD is very important, because it can lead to serious complications, especially cardiovascular adverse events. Atypical or incomplete KD diagnosis is challenging. Uncommon clinical feature of KD in children have been reported in previous case reports including pyuria, meningitis, ventricular arrhythmia, or shock [12, 13]. Introducing such atypical presentations of KD will be helpful in increasing physicians' attention to suspicious KD and preventing delayed diagnosis. In the case we mentioned here, a seven‐year‐old girl presented with initial isolated torticollis, and subsequently, other classic symptoms were added; finally, a giant aneurysm was diagnosed.

Incomplete KD defined in patients with fever lasting five or more days and with two or three of the classical findings (exanthema, conjunctivitis, edema in the limbs, erythema of oral mucosa and lips, cervical lymphadenopathy). The true incidence of this type of KD is difficult to determine [14, 15]. However, the definition “atypical KD” should be considered for patients with clinical presentation including renal involvement, unilateral peripheral facial nerve palsy, testicular swelling, pulmonary infiltrations, pleural effusions, diarrhea, vomiting, and abdominal pain, acute abdomen, hemophagocytic syndrome that routinely are not categorized in KD [16].

Torticollis in this case seemed to be due to painful cervical lymphadenitis [17]. The rate of cervical lymphadenopathy occurring as the initial presenting symptoms is approximately 12% [18]. Despite the uncommon manifestation, some cases have been reported previously of KD presented by torticollis. Dyer et al. reported a 6‐year‐old male who presented with torticollis. In their case, a series of investigations for elevated inflammatory markers revealed dilated coronary artery aneurysms on echocardiogram, and he was treated with high‐dose IVIG and low‐dose aspirin [17]. Runel‐Belliard et al. reported a case of KD with arthritis in a 4‐year‐old girl whose initial presentation was a febrile torticollis [19]. In another case, a nine‐year‐old boy presented with fever, lymphadenopathy, parotitis, and torticollis with an initial diagnosis of infective parotitis, and finally, KD was diagnosed [20]. In our case, she underwent CABG because of LV dysfunction, and after that, she was followed and was healthy until 2 years later.

Dilatation of the coronary arteries can occur in approximately 10%–20% of KD patients. In half of them, the aneurysms can regress within 1 or 2 years, and no abnormalities are found by angiography in the coronary‐arterial system. In the remaining patients, in contrast, the aneurysms can persist with obviously irregular lumens of the coronary arteries. In 3% of the patients initially having aneurysms, coronary‐arterial obstruction progresses. The time span between the onset of the disease and development of the coronary‐arterial stenosis leading to CABG varies from several months to 20 years. The indications for CABG should be determined not only by angiographic findings but also by other clinical factors, such as severity of myocardial ischemia, history of myocardial infarction, and ventricular performance [21, 22, 23, 24, 25]. It is very difficult to determine the indications for catheter intervention and CABG in children based on the diagnosis and localization of the affected coronary artery, using standard methods [26].

The main finding of this case was a very atypical presentation of silent KD presented by torticollis. The patient had no initial manifestations of KD; the diagnosis was not based on criteria, and it was out of it. So, in isolated torticollis, considering KD diagnosis in children as atypical KD should be emphasized beside other differential diagnoses.

## Author Contributions

**Hassan Mottaghi Moghaddam Shahri:** conceptualization, data curation, investigation, supervision, writing – original draft, writing – review and editing. **Faeze Keihanian:** data curation, writing – original draft, writing – review and editing. **Mohammad Hassan Nezafati:** investigation, supervision, writing – review and editing. **Mohammad Saeid Sasan:** data curation, investigation, writing – review and editing.

## Ethics Statement

This study was performed in accordance with the Helsinki declaration. Data were published anonymously.

## Consent

A written informed consent was obtained from the patient.

## Conflicts of Interest

The authors declare no conflicts of interest.

## Data Availability

Data sharing is not applicable to this article, as no datasets were generated or analyzed during the current study.
